# Cardiovascular Risk Factors and Distributions of the Ankle-Brachial Index among Type 2 Diabetes Mellitus Patients

**DOI:** 10.1155/2012/485812

**Published:** 2012-08-05

**Authors:** Badaruddoza Doza, Manpreet Kaur, Sonia Chopra, Rohit Kapoor

**Affiliations:** ^1^Department of Human Genetics, Guru Nanak Dev University, Punjab, Amritsar 143005, India; ^2^Carewell Heart and Super Speciality Hospital, Amritsar, India

## Abstract

*Background*. The aim of present study is to observe the association between the levels of ankle-brachial index (ABI) and cardiovascular risk factors among people with type 2 diabetes mellitus in north India. A cross-sectional study was carried out at a centre for heart and diabetic clinic in the state of Punjab on 1121 subjects (671 males and 450 females) with type 2 diabetes mellitus. History of symptoms related to cardiovascular diseases was noted, and blood pressure and anthropometric measurements were recorded. Ankle-brachial index (ABI) was measured using ultrasonic Doppler flow detector. Subjects with ABI ≤0.9 and ≥1.30 were classified as having low and high ABI, respectively. Females had a higher BMI and brachial-ankle pulse wave velocity (*P* < 0.001). Whereas, males had higher diastolic blood pressure and duration of type 2 diabetes mellitus. The differences of systolic blood pressure and ankle-brachial index were not found significant between the sexes. The prevalence of low ABI (<0.9) was 4.47% in men and 4.67% in women and high ABI (≥1.30) was prevalent in 14% of men and 10.45% of women. Age, BMI, baPWV, and blood pressures were significantly associated with ABI value in both sexes. The results suggested that the ABI might be used as a strong indicator for cardiovascular risk factors in type 2 diabetic subjects.

## 1. Introduction

It is well known that ankle-brachial index (ABI) is simple and useful method to assess the peripheral vascular diseases and it is also widely used in clinical and epidemiological studies [1–3]. Ankle-brachial index (ABI) is the ratio of the resting ankle to brachial systolic blood pressure. A low ABI (<0.90) was considered to be a predictor for risk of cardiovascular diseases [3, 4]. The measurement of ABI is also recommended by American Heart Association as a diagnostic criterion for the prevalence of peripheral arterial diseases [5]. However, many population-based association studies between ABI distribution and cardiovascular diseases in type 2 diabetes have been carried out in Western countries, whereas, no such epidemiological data are available in north Indian subjects with type 2 diabetes mellitus till date according to the information gathered from pubmed. Therefore, we conducted a hospital-based study to document the association between distribution of the ankle-brachial index and cardiovascular risk factors in the north Indian subjects diagnosed with type 2 diabetes mellitus in order to provide the baseline data for further prospective study.

## 2. Material and Methods

### 2.1. Study Design

This study recruited 1121 type 2 diabetic patients (671 male and 450 female). The diagnosis of type 2 diabetes was made according to the criteria of World Health Organization. The subjects for this cross-sectional study were selected consecutively from the population coming to the centre named “Heart Station and Diabetes Clinic” under Care Well Heart Super-speciality Hospital situated in Amritsar city in the State of Punjab. All the patients were collected from the same centre to avoid the heterogeneity of the patients and investigators. The exclusion criteria were subjects with type 1 diabetes, less than 40 years old, those with amputation of both lower limbs, and taking vasodilators.

### 2.2. Anthropometric Measurements

The height was measured using anthropometric rod with subject standing in erect position with the head in the ear-eye plane. The reading was then recorded to the nearest 0.1 cm. The weight of the subject was measured in kilogram by making him/her stand on a weighing machine with light clothing and without shoes. Weight was recorded with an allowance deducted for clothing to the nearest 0.5 kg. The body mass index (BMI) was calculated as weight divided by height squared (kg/m^2^). The age of the subjects was determined directly from their reported date of birth.

### 2.3. Measurements of Blood Pressure

Two consecutive readings were recorded for each systolic and diastolic blood pressure (SBP and DBP), and the average was used. The measurements were taken with the help of mercury sphygmomanometer in a sitting position with the right forearm placed horizontal on the desk as recommended by the American Society of Hypertension [6]. The first appearance (phase I) and disappearance (phase V) of Korotkoff's sound were used to define systolic and diastolic blood pressure.

### 2.4. Measurements of the Ankle-Brachial Index (ABI)

The ABI was measured according to a standardized protocol [2] as the ratio of ankle systolic blood pressure to brachial systolic blood pressure using a bidirectional automatic Doppler ultrasound (VP-2000/1000-Colin Corporation, Hyayashi Komaki, Japan) ABI was calculated by using the higher of the two brachial pressures as denominator and higher of the two ankle pressures as numerator. The ABI values were classified into 6 categories such as: <0.90, 0.90–0.99, 1.00–1.09, 1.10–1.19, 1.20–1.29, and >1.30. An ABI value less than 0.90 and higher than 1.3 were considered low and high, respectively. 

### 2.5. Measurements of Brachial-Ankle Pulse Wave Velocity (baPWV)

Blood pressure and baPWV were measured using an automated wave form analyser. Blood pressure was measured in both arms and the average of both left and right baPWV measurements were used for further analysis.

### 2.6. Statistical Analysis

Data are expressed as the mean ± SD according to sex. The comparison of mean differences of clinical characteristics between males and females were analyzed by Student's *t*-test. The differences of age-adjusted mean values of cardiovascular risk factors according to ABI categories were calculated using an analysis of covariance (ANCOVA). Correlation was calculated by regression analysis. The *P* < 0.05 level was selected as the criterion of the statistical significance. All statistical analyses were performed using SPSS software 17.0 version (SPSS, Chicago, IL, USA).

## 3. Results

The comparison of clinical features between men and women with type 2 diabetes mellitus of the study population is presented in Table 1. The age, BMI, and brachial-ankle pulse wave velocity (baPWA) were significantly higher (*P* < 0.001) in women than in men. The diastolic blood pressure (DBP), duration of type 2 diabetes mellitus since diagnosis were significantly (*P* < 0.001) higher in men than in women. However, the differences of systolic blood pressure (SBP) and ankle-brachial pressure index (ABI) were not significant between the sexes. The percentage of hypertension was marginally higher in women (62.88%) as compared to men (62.58%).

The distributions of ABI according to sex with other clinical characteristics are presented in Tables 2 and 3. In the most subjects (28% for men and 32% for women), ABI was between 1.10–1.19 and 1.00–1.09 for men and women respectively, followed by 1.00–1.09 (23%) and 0.90–0.99 (16%) for men and 1.10–1.19 (29%) and 1.20–1.29 (13%) for women. The prevalence of low ABI (<0.90) was 4.47% in men and 4.67% in women and a high ABI (≥1.30) was observed to be 14% and 10.45% in men and women respectively. The overall differences of means in different categories of ABI distribution have been found significant for BMI, SBP, and baPWV for both sexes (*P* < 0.001) and age, DBP, and duration of type 2 diabetes in men (*P* < 0.001). The measurements of baPWV have not been taken from the patients with low (<0.90) and high (>1.30) ABI due to unreliable assessments. Most of the hypertensive individuals with type 2 diabetes mellitus (17.88% and 20.89%) were found in ABI values in men and women in the range 1.10–1.19 and 1.00–1.09, respectively. The highest mean values of BMI, SBP and DBP (29.12 ± 5.34, 173.00 ± 22.63, and 93.93 ± 14.12 resp.) were observed in low ABI (<0.90) in men whereas, the highest mean values of BMI (30.45 ± 5.27) and SBP (160.33 ± 28.26) in low ABI (<0.90) were observed in women. The highest mean values of baPWV in both men and women were observed in between 1.20–1.29 ABI level (1992.49 ± 515.46 for men and 2079.20 ± 567.25 for women, resp.).

In all subjects with type 2 diabetes (Table 4) ABI was negatively correlated with SBP (*r* = −0.247, *P* = 0.001; *r* = −0.145, *P* = 0.002) and DBP (*r* = −0.171, *P* = 0.001; *r* = −0.102, *P* = 0.029) for men and women, respectively. The BMI was also negatively correlated in men only (*r* = −0.296, *P* = 0.001). However, brachial-ankle pulse wave velocity (baPWV) were significantly correlated with ABI (*r* = 0.181, *P* = 0.001) in men but not in women. It is also observed that age is significantly correlated in both men and women (*r* = 0.164, *P* = 0.001 and *r* = 0.22, *P* = 0.001 for men and women, resp.). We have found no significant correlation between duration of type 2 diabetes and ABI in both sexes. To determine independent influences and association of individual clinical factors on ABI, we performed a multiple logistic regression analysis for both sexes (Table 5). A significant inverse association was observed between BMI (OR: 0.714, 95% CI 0.657–0.774, *P* = 0.001), SBP (OR: 0.977, 95% CI 0.964–0.991, *P* = 0.001), and duration of type 2 diabetes (OR: 0.984, 95% CI 0.952–1.017, *P* = 0.05) with ABI in men, whereas, BMI showed significant positive association (OR: 1.51, 95% CI 1.352–1.687, *P* = 0.001) with ABI in women. A significant positive association of age (OR: 1.49, 95% CI 0.880–2.22, *P* = 0.01) with ABI has been observed in men, but in women age has no association in women (OR: 0.991, 95% CI 0.961–1.023, *P* = 0.589). No associations have been observed between DBP and baPWV with ABI in both sexes.

## 4. Discussion

The present study demonstrated that prevalence of low ABI was 4.47% in men and 4.67% in women with type 2 diabetes mellitus. These values are almost similar with one Japanese study (5%) [7] and higher than a Korean population study (2.2% in men and 1.8% in women) [3]. Despite the low prevalence of low ABI values in the present study, type 2 diabetic individuals with low ABI were significantly associated with obesity (29.12 ± 5.34) and cardiovascular risk factors (SBP: 173 ± 22.63 mmHg; DBP: 93.93 ± 14.12 mmHg) in men, but in women it is associated with only SBP (160.33 ± 28.26 mmHg). Therefore, low ABI <0.9 was in a significant association with cardiovascular disease risk. In type 2 diabetic patients, baPWV showed maximum when ABI values were greater than 1.20 for both men and women. The phenomena are related with the severity of cardiovascular disease. Therefore, the data implicated that baPWV combined with the range of ABI values in type 2 diabetic patients could act as a screen and diagnosis factor for peripheral artery diseases. The results are also supported by few studies in Chinese, Korean, and Brazilian populations [8–11]. The study demonstrated that in patients with type 2 diabetes, SBP and DBP showed significant negative correlations with ABI in both sexes. We also found negative correlation between BMI and ABI in men but not in women. These negative correlations further strengthen the hypothesis that a low ABI ≤0.9 is a useful diagnostic tool for detecting peripheral vascular diseases and would also be considered as a strong predictor for cardiovascular morbidity and mortality in type 2 diabetic patients. However, the present study also showed significant correlation between baPWV and ABI in men but not in women. This showed we could not assess the arterial calcification with respect to ABI values among type 2 diabetic patients. A longer duration of type 2 diabetes is independently associated with low ABI in both sexes. The higher prevalence of hypertension has been found for ABI between 1.10–1.19 in both the sexes. Similar to these results, many other studies have also reported that a low ABI and high ABI are associated with the increased risk of cardiovascular events [12–15]. There is a strong association of increasing age with ABI, especially in men. It has also been reported [16, 17] that prevalence of peripheral arterial disease is increasing in adults aged >40 years on the basis of mean ankle-brachial index <0.9 in India and USA.

### 4.1. Certain Limitations of the Present Study Should Be Counted

The cross-sectional nature of the study design, which cannot provide cause and effect relationship. A prospective study should be undertaken to establish the relationship between ABI distribution and development of cardiovascular diseases. The study was based on self-reporting of the duration of diabetes. There were no medical records available for verifications. Another limitation was lack of data of many baseline characteristics such as smoking, drinking, dyslipidemia, and family history. All subjects in the study were confirmed diabetic since many years; therefore, in present time no glucose tolerance test has been done to any subjects. 

## 5. Conclusion 

The present results revealed positive and significant associations between low-ABI values and cardiovascular risk factors. Therefore, a low-ABI value may be a useful marker and powerful predictor for cardiovascular diseases. However, a prospective further study would be required for more confirmation of the hypothesis.

## Figures and Tables

**Table 1:** Clinical characteristics of the subjects with type 2 diabetes mellitus.

Variables	Men (*n* = 671)	Women (*n* = 450)	^ ∗^ *P* value
	Mean ± S.D	Mean ± S.D	
Age (yrs)	52.97 ± 10.04	54.76 ± 8.81	<0.002
Body mass index (kg/m^2^)	25.71 ± 5.15	26.66 ± 4.94	<0.002
Systolic blood pressure (mmHg)	150.12 ± 23.48	150.32 ± 24.87	0.890
Diastolic blood pressure (mmHg)	87.67 ± 13.81	83.86 ± 12.04	<0.001
Ankle-brachial pressure index (ABI)	1.13 ± 0.17	1.12 ± 0.14	0.293
Brachial-ankle pulse wave velocity (baPWV) (cm/sec)	1905.13 ± 478.33	1991.40 ± 522.08	<0.004
Duration (yrs)	8.02 ± 5.64	6.96 ± 5.54	<0.002
Hypertension (%)	62.59	62.88	—

^∗^ *P* value by *t*-test; duration: since diagnosis of type 2 diabetes mellitus.

**Table 2:** Age-adjusted means and standard deviations of cardiovascular risk factors according to ABI levels among men (*n* = 671) with type 2 diabetes mellitus.

Variables	Ankle-brachial index (ABI)						
	<0.90 (*n* = 30)	0.90–0.99 (*n* = 106)	1.00–1.09 (*n* = 155)	1.10–1.19 (*n* = 185)	1.20–1.29 (*n* = 101)	≥1.30 (*n* = 94)	^ ∗^ *P* value for overall difference
Number (%)	4.47	15.80	23.10	27.57	15.05	14.01	—
Age (yrs)	49.27 ± 12.43	50.83 ± 11.38	51.25 ± 8.50	55.12 ± 10.20	53.96 ± 9.58	54.78 ± 9.52	<0.001
BMI (kg/m^2^)	29.12 ± 5.34	27.58 ± 4.69	26.28 ± 4.52	25.58 ± 4.81	23.97 ± 4.37	22.73 ± 5.33	<0.001
SBP (mmHg)	173.00 ± 22.63	154.10 ± 24.22	153.52 ± 24.96	148.82 ± 21.54	148.71 ± 23.25	139.99 ± 17.22	<0.001
DBP (mmHg)	93.93 ± 14.12	90.28 ± 15.98	89.40 ± 15.26	86.89 ± 12.38	87.97 ± 12.41	82.60 ± 11.35	<0.001
baPWV (cm/sec)	—	1761.11 ± 413.77	1874.88 ± 440.24	1929.36 ± 495.17	1992.49 ± 515.46	—	<0.001
Duration (yrs)	8.23 ± 6.54	5.80 ± 4.68	8.01 ± 6.08	9.17 ± 7.49	8.14 ± 6.64	8.32 ± 6.91	<0.003
Hypertension (%)	4.47	10.88	15.20	17.88	9.54	6.41	—

^∗^ *P* value of age adjusted analysis of covariances (ANCOVA); baPWV: Brachial-ankle pulse wave velocity; BMI: Body mass index; SBP: Systolic blood pressure; DBP: Diastolic blood pressure; duration: since diagnosis of type 2 diabetes mellitus.

**Table 3:** Age-adjusted means and standard deviations of cardiovascular risk factors according to ABI levels among women (*n* = 450) with type 2 diabetes mellitus.

Variables	Ankle-brachial index (ABI)						
	<0.90 (*n* = 21)	0.90–0.99 (*n* = 44)	1.00–1.09 (*n* = 146)	1.10–1.19 (*n* = 132)	1.20–1.29 (*n* = 60)	≥1.30 (*n* = 47)	^ ∗^ *P* value for overall difference
Number (%)	4.67	9.78	32.44	29.33	13.33	10.45	—
Age (yrs)	57.48 ± 10.39	54.23 ± 8.40	53.95 ± 9.13	55.48 ± 7.81	56.03 ± 9.33	53.06 ± 8.77	0.198
BMI (kg/m^2^)	26.52 ± 4.87	26.74 ± 4.72	25.98 ± 5.51	25.71 ± 3.62	27.47 ± 4.57	30.45 ± 5.27	<0.001
SBP (mmHg)	160.33 ± 28.26	152.98 ± 21.57	153.10 ± 27.22	149.94 ± 21.21	145.50 ± 23.65	143.77 ± 20.27	<0.034
DBP (mmHg)	83.95 ± 12.93	85.07 ± 9.07	85.08 ± 13.25	83.96 ± 9.73	80.45 ± 9.95	81.04 ± 11.17	0.064
baPWV (cm/sec)	—	1855.64 ± 368.47	1996.37 ± 488.16	2046.62 ± 493.25	2079.20 ± 567.96	—	<0.001
Duration (yrs)	8.20 ± 7.88	6.17 ± 5.17	6.55 ± 5.34	7.39 ± 5.28	7.68 ± 5.64	7.40 ± 5.93	0.462
Hypertension (%)	3.56	7.56	20.89	19.56	6.89	5.56	—

^∗^ *P* value of age-adjusted analysis of covariance (ANCOVA); baPWV: Brachial ankle pulse wave velocity; BMI: Body mass index; SBP: Systolic blood pressure; DBP: Diastolic blood pressure; duration: since diagnosis of type 2 diabetes mellitus.

**Table 4:** Univariate analysis of relationships between ABI and characteristics of subjects with type 2 diabetes mellitus.

Variables	Men		Women	
	*r*	*P* value	*r*	*P* value
Age (yrs)	0.164	0.001	0.22	<0.001
BMI (kg/m^2^)	−0.296	0.001	0.178	<0.001
SBP (mmHg)	−0.247	0.001	−0.145	<0.002
DBP (mmHg)	−0.171	0.001	−0.102	<0.029
baPWV (cm/sec)	0.181	0.001	0.027	0.563
Duration (yrs)	0.065	0.091	0.050	0.280

baPWV: Brachial-ankle pulse wave velocity; BMI: Body mass index; SBP: Systolic blood pressure; DBP: Diastolic blood pressure; duration: since diagnosis of type 2 diabetes mellitus.

**Table 5:** Multiple logistic regression analysis of various clinical factors and ankle-brachial pressure index (ABI).

Factors	Men			Women		
	Odds ratio	95% confidence interval	*P* value	Odds ratio	95% confidence interval	*P* value
Age (yrs)	1.40	0.880–2.22	0.010	0.991	0.961–1.023	0.589
BMI (kg/m^2^)	0.714	0.657–0.774	0.001	1.510	1.352–1.687	0.001
SBP (mmHg)	0.977	0.964–0.991	0.001	0.986	0.966–1.007	0.190
DBP (mmHg)	0.995	0.972–1.018	0.671	0.996	0.960–1.034	0.845
baPWV (cm/sec)	1.001	1.000–1.003	0.559	1.001	0.999–1.003	0.190
Duration (yrs)	0.984	0.952–1.017	0.05	0.956	0.913–1.000	0.05

baPWV: Brachial-ankle pulse wave velocity; BMI: Body mass index; SBP: Systolic blood pressure; DBP: Diastolic blood pressure; duration: since diagnosis of type 2 diabetes mellitus.
